# The Basement Membrane in a 3D Breast Acini Model Modulates Delivery and Anti-Proliferative Effects of Liposomal Anthracyclines

**DOI:** 10.3390/ph13090256

**Published:** 2020-09-19

**Authors:** Tabea Wiedenhoeft, Tobias Braun, Ronald Springer, Michael Teske, Erik Noetzel, Rudolf Merkel, Agnes Csiszár

**Affiliations:** Forschungszentrum Jülich, Institute of Biological Information Processing (IBI-2), Mechanobiology, 52428 Jülich, Germany; t.wiedenhoeft@fz-juelich.de (T.W.); tobi.braun@gmx.de (T.B.); r.springer@fz-juelich.de (R.S.); michael@mictes.de (M.T.); e.noetzel@fz-juelich.de (E.N.); r.merkel@fz-juelich.de (R.M.)

**Keywords:** fusogenic liposomes, PEGylated liposomes, doxorubicin delivery, lysosomal trapping, basement membrane

## Abstract

Breast cancer progression is marked by cancer cell invasion and infiltration, which can be closely linked to sites of tumor-connected basement membrane thinning, lesion, or infiltration. Bad treatment prognosis frequently accompanies lack of markers for targeted therapy, which brings traditional chemotherapy into play, despite its adverse effects like therapy-related toxicities. In the present work, we compared different liposomal formulations for the delivery of two anthracyclines, doxorubicin and aclacinomycin A, to a 2D cell culture and a 3D breast acini model. One formulation was the classical phospholipid liposome with a polyethylene glycol (PEG) layer serving as a stealth coating. The other formulation was fusogenic liposomes, a biocompatible, cationic, three-component system of liposomes able to fuse with the plasma membrane of target cells. For the lysosome entrapment-sensitive doxorubicin, membrane fusion enabled an increased anti-proliferative effect in 2D cell culture by circumventing the endocytic route. In the 3D breast acini model, this process was found to be limited to cells beneath a thinned or compromised basement membrane. In acini with compromised basement membrane, the encapsulation of doxorubicin in fusogenic liposomes increased the anti-proliferative effect of the drug in comparison to a formulation in PEGylated liposomes, while this effect was negligible in the presence of intact basement membranes.

## 1. Introduction

Breast cancer occurrence is rather well monitored by the mammographic screening of females in the European Union [1]. As the long-term survival is increasing in accordance with early diagnosis, incidence mainly plateaued over the last decade. Therefore, breast cancer is still the most common female cancer by far, with second-highest mortality [2,3]. For decades, anthracyclines have been an integral part of commonly used regimens in adjuvant and non-targeted therapy, decreasing the 10-year risk of breast cancer recurrence and overall mortality [4]. Nevertheless, the use of anthracyclines in cancer therapy increases the risk of adverse effects like therapy-related toxicities including cardiomyopathy [5,6].

To improve the safety profile, doxorubicin formulation was optimized by encapsulation in polyethylene glycol (PEG)ylated liposomes that led to the approval of Doxil^®^. This liposomal drug formulation has markedly reduced the risk of doxorubicin-induced cardiotoxicity [7,8]. Though PEGylation of liposomes increased extravasation to the tumor site by passive targeting, adverse effects could be observed as well, e.g., due to liposome-induced complement activation and hypersensitivity reactions [9,10]. To this date, extensive research has been investigating different formulation strategies to reduce adverse side effects of the drug and enhance its therapeutic efficacy, including the application of polymer nanoparticle and conjugates, exosome incorporation, prodrug self-assembly, and intercalation into oligonucleotides [11,12,13,14,15]. However, no non-liposomal drug delivery system of doxorubicin is approved so far.

In this study, we compared two different lipid-based formulations of anthracyclines for their uptake into cells and their capacity to pass basement membranes. These formulations were established PEGylated endocytic liposomes (PEG-EL) and fusogenic liposomes (FL). The latter have been previously described as biocompatible [16,17] three-component-systems comprised of a positively charged and a neutral lipid as well as an aromatic compound. These lipid particles fuse with the plasma membrane of mammalian cells via a protein-independent mechanism [18,19]. The uptake mechanism of cargo is highly efficient with limited degradation and so far has been described for proteins, small molecules, and nucleic acids in vitro and in vivo [17,20,21]. In contrast, the cellular uptake of conventionally used liposomes is mediated by endocytosis [22], facilitating lysosomal sequestration and thereby the elimination of the delivered cargo.

**Figure 1:**
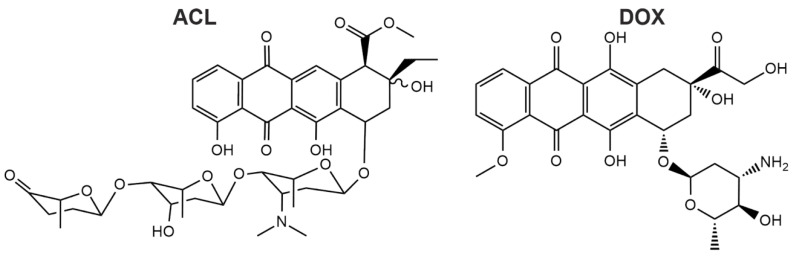
Chemical structures of aclacinomycin A (ACL) and doxorubicin (DOX) are shown. ACL has a more complex structure compared to DOX due to the long aglycolic side chain.

Although the increased cellular uptake and accumulation of drugs could be mediated by membrane fusion, the application of these lipid- and lipid derivative-based fusogenic liposomes in cancer therapy is missing, yet cancer therapy is the largest application field of liposome-based drugs in clinical use [23]. Consequently, we investigated the delivery of the model substances doxorubicin (DOX) and aclacinomycin A (ACL) (Figure 1), also called aclarubicin, using FL and PEG-EL and compared their pharmacological effects to those of the free drug.

In the organism, breast cancer development starts with the transformation of epithelial cells. These are supported by a basement membrane that forms an important physiological barrier against nano-therapeutics and cancer cell invasion or infiltration. The extent of basement membrane damage is a crucial criterion for cancer classification and treatment prognosis [24]. While the in vitro investigation in two-dimensional (2D) cell culture is a necessity for the initial screening of drug candidates, a more sophisticated, three-dimensional (3D) model involving the basement membrane might be an additional, more stringent approach to testing the suitability of a drug candidate or formulation. The cytotoxic effects of anthracyclines in classical liposomal formulations have been investigated in tumor spheroids before [25,26,27]. In this study, we used a 3D acini model of the mammary gland with altering integrity of the basement membrane to quantify the delivery efficiency of DOX and ACL, two anthracyclines, using liposomal carriers taken up by membrane fusion (FL) or endocytosis (PEG-EL).

## 2. Results

### 2.1. Characterization of FL and PEG-EL Loaded with ACL and DOX

We investigated the suitability of FL for the delivery of anti-cancer therapeutics in comparison to a liposomal drug formulation similar to liposomes already used in cancer therapy [7,28]. These liposomes resemble pharmacologically relevant PEGylated liposomes and will be further referred to as PEG-EL based on their entry mechanism into mammalian cells. FL and PEG-EL were both loaded with DOX and ACL. Both compounds share a planar anthraquinone scaffold linked to differing amino sugar moieties. The more complex oligosaccharide of ACL increases lipophilicity [29] (Figure 1). First, the size and zeta potential of the two liposomal formulations were compared. In all cases, the mean size was in the range of 80 to 165 nm (Table 1). The zeta potential of FL was positive and not dependent on cargo addition. Due to the contribution of PEGylated lipids, the phosphatidylcholine-based PEG-EL formulation exhibited negative zeta potentials, not significantly changed by the addition of anthracyclines (Table 1). The encapsulation efficiencies of ACL and DOX were analyzed by filtration and subsequent fluorescence spectroscopy. They corresponded to approximately 90% in all liposomal formulations (Table 1); therefore, we used both formulations without further purification in the remainder of this study.

**Figure 2:**
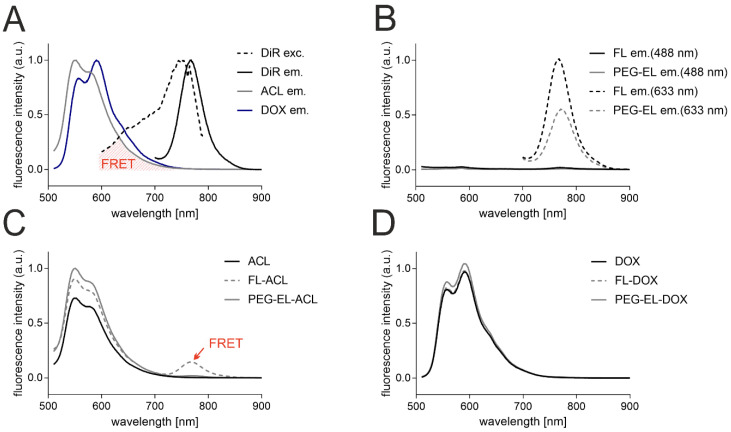
Analysis by fluorescence spectroscopy of the anthracyclines and the membrane tracer dye DiR indicated a spectral overlap of anthracyclines’ fluorescence emission (ACL em. and DOX em.) and DiR excitation (DiR exc.) suitable for Foerster Resonance Energy Transfer (FRET) (**A**). Fluorescence intensity was normalized with respect to the individual highest fluorescence intensity for better visualization. DiR in the liposomal formulations of FL and PEG-EL showed a noticeable fluorescence emission upon excitation at 633 nm but not at 488 nm in the given experimental setting (**B**). Data were normalized with respect to the highest fluorescence intensity of DiR emission in FL upon excitation at 633 nm. Incorporation of ACL in FL resulted in possible DiR emission after excitation at 488 nm due to FRET (**C**). A similar FRET signal was not observed when FL were loaded with DOX (**D**). In C and D, data were normalized with respect to the highest fluorescence intensity of anthracycline emission in PEG-EL. Data are shown as normalized mean curves; a DiR excitation spectrum is shown representatively.

Further, to determine the anthracycline localization in the liposomal formulations, the interaction of the anthracyclines with the liposomal bilayer-incorporated membrane dye DiR was investigated with fluorescence spectroscopy. The emission of ACL and DOX upon excitation at 488 nm peaked between 550 and 600 nm, and the emission of DiR upon excitation at 633 nm peaked at 770 nm. The excitation spectrum of DiR was recorded between 600 and 790 nm, while the emission spectrum of DiR did not overlap with those of anthracyclines (Figure 2A). Hence, the spectral overlap of the emission of anthracyclines and the excitation of the membrane dye DiR could lead to measurable Foerster resonance energy transfer (FRET) if membrane dye and ACL/DOX were in close proximity. The excitation of DiR in the liposomal formulations FL and PEG-EL at 633 nm did led to an emission peak as observed before, while no significant emission could be detected after excitation at 488 nm without anthracycline addition (Figure 2B). Next, the emission spectra of the anthracyclines ACL and DOX in the liposomal formulations FL and PEG-EL and as a free drug in PBS were investigated. The emission of DOX and ACL in different formulations peaked again between 550 and 600 nm, while the formulation of ACL in fusogenic liposomes (FL-ACL) showed a small additional peak at 770 nm, demonstrating FRET of the ACL fluorophore to DiR (Figure 2C). No FRET peak was observed for the more polar DOX in any formulation (Figure 2D).

### 2.2. Uptake Efficiency of ACL and DOX into MCF-10A Cells in 2D Cell Culture

**Figure 3:**
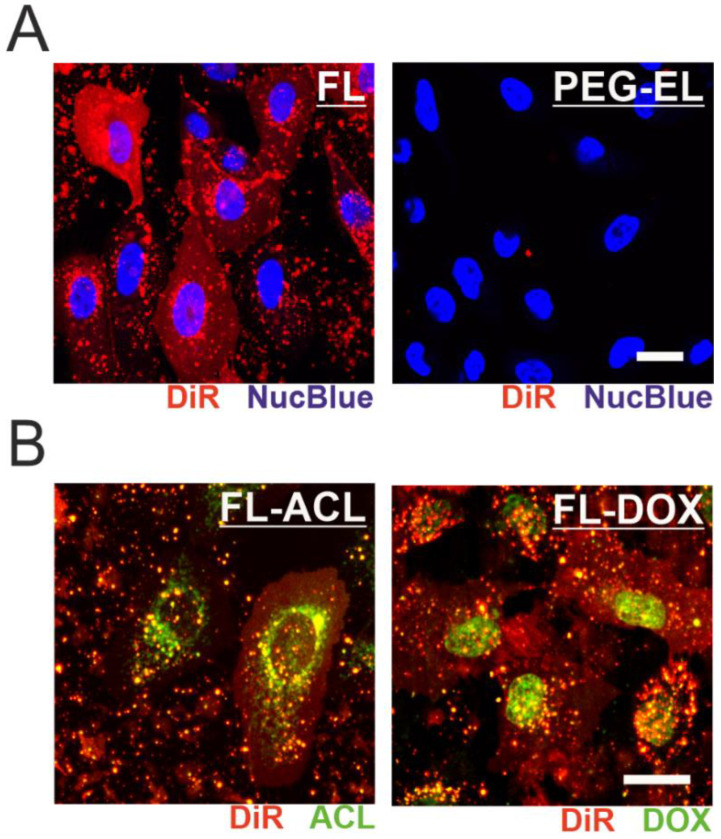
Monitoring the cellular uptake of anthracycline-loaded liposomal formulations. Fusion of FL with MCF-10A cell membrane was observed by incorporation of DiR, leading to a homogenous membrane staining not observed after treatment with PEG-EL (**A**). Incorporation of ACL (FL-ACL) or DOX (FL-DOX) did not affect membrane fusion, yet subcellular localization of ACL and DOX differed after cellular uptake (**B**). Scale bars 20 µm.

We further investigated the uptake mechanism of the two liposomal formulations by monitoring the far-red membrane tracer dye signal. After treatment of MCF-10A cells with FL, the intercalation of DiR into the plasma membrane was demonstrated by a fairly homogenous plasma membrane staining indicating membrane fusion, while treatment with PEG-EL did not result in the spread of DiR within the plasma membrane (Figure 3A). ACL and DOX were effectively delivered into MCF-10A cells by membrane fusion of the FL formulation, as observed by confocal microscopy. Moreover, loading of FL with ACL or DOX to a final concentration of 6 µM or 9 µM, respectively, did not alter the uptake mechanism of FL into mammalian cells (Figure 3B). Within the cell, DOX was mostly localized in the cell nucleus, whereas ACL was enriched in a substructure of the cell that enclosed the nucleus (Figure 3B). This difference in localization was independent of anthracycline formulation (Supplemental Figure S1) and points towards possible differences in the mechanism of action of the two compounds.

**Figure 4:**
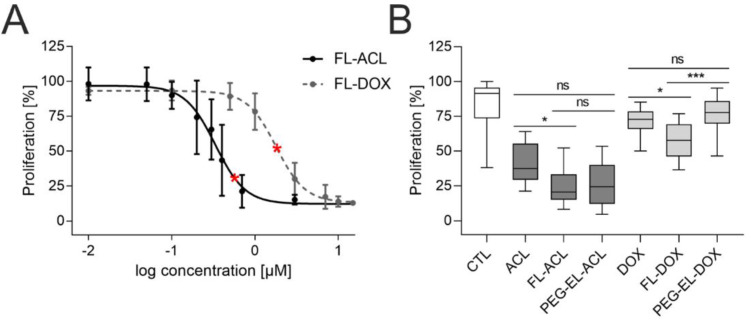
Anti-proliferative effect of anthracyclines in MCF-10A cells. Investigation of the proliferation of MCF-10A cells by cell count using flow cytometry demonstrated a dose-dependent anti-proliferative effect of both ACL and DOX encapsulated in FL (**A**). The anti-proliferative effect of ACL and DOX was affected by the formulation, with significantly increasing potency for FL-ACL and FL-DOX in comparison to the free drugs and for FL-DOX in comparison to PEG-EL-DOX in an EdU (5-ethynyl-2′-deoxyuridine) incorporation assay (**B**). Samples treated with PBS served as a control (CTL). The used doses of ACL and DOX in the EdU incorporation assay were 0.6 µM and 1.8 µM, respectively, and are indicated by red stars in (**A**). Mean with standard deviation (**A**) and box plots (**B**) are shown. Statistical evaluation was done using non-parametrical Kruskal–Wallis test with post-hoc Dunn’s test (*: *p* < 0.05; ***: *p* < 0.0005).

To investigate the anti-proliferative effect of ACL and DOX, we varied the final concentration of anthracycline in FL in a range from 0.01 µM to 15 µM and counted cells 72 h post-treatment. Similar doses of FL-ACL led to a stronger proliferation reduction in comparison to FL-DOX (Figure 4A); this was observed also after incorporation in PEG-EL and for the free drug (Supplemental Figure S2).

For further comparison of the anthracyclines as free drugs or in both liposomal formulations, an EdU (5-ethynyl-2′-deoxyuridine) incorporation assay was used. All formulations besides PEG-EL-DOX led to a significantly lower proliferation. Treatment with FL-DOX decreased proliferation significantly more than treatment with PEG-EL-DOX. The uptake of DOX using membrane fusion of FL, therefore, seemed to increase the intracellular availability of the drug by circumventing endocytosis (Figure 4B). FL-ACL also reduced proliferation significantly if compared to the free drug.

### 2.3. Influence of Basement Membrane Integrity on Liposomal Uptake Efficiency

**Figure 5:**
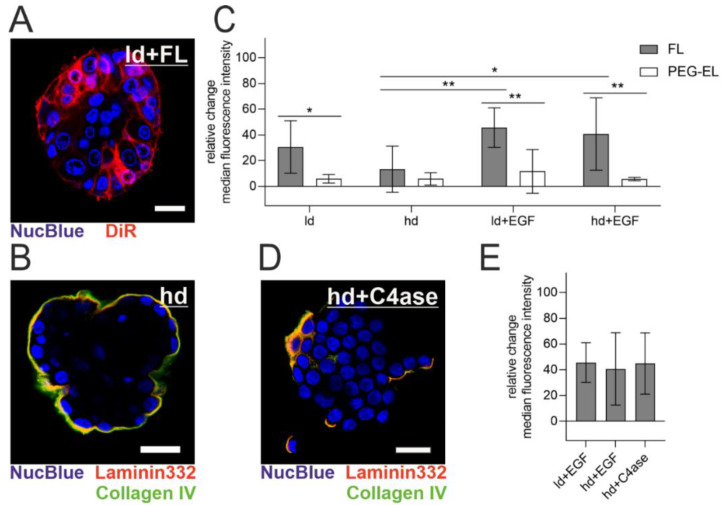
Liposomal uptake into MCF-10A cells in a 3D acini model. Note the high extent and homogeneity of plasma membrane staining with DiR in acini with a lowly developed (ld) basement membrane (BM) (**A**). The highly developed (hd) BM of MCF-10A acini was imaged by immunofluorescent staining of collagen IV and laminin 332 (**B**). The relative change of median fluorescence intensity of DiR incorporated in cells cultured in the MCF-10A acini model was investigated using flow cytometry after treatment with FL or PEG-EL (**C**). A highly developed BM could be degraded by treatment with collagenase IV, as be observed by immunofluorescent staining of collagen IV and laminin 332 (**D**). Degradation of the BM before treatment with FL led to a relative change of median fluorescence intensity comparable to that of acini having a compromised BM (**E**). Scale bars 20 µm. Mean with confidence interval (95%) is shown, statistical evaluation was done using one-way ANOVA with post-hoc Tukey’s test (*: *p* < 0.05; **: *p* < 0.01).

The MCF-10A acini model enables the investigation of polarized epithelial cells protected by a basement membrane (BM) with modular maturation and integrity: the meshwork of BM-associated proteins increases in thickness with cultivation, while additional treatment with Epidermal Growth Factor (EGF) induces acinar differentiation loss, manifested in our model by a higher cell count [30,31]. The BM was visualized by immunofluorescent staining of its two key components collagen IV and laminin 332 (Figure 5B). When FL were applied to MCF-10A acini with a lowly developed (ld) BM (10 days of culturing time), a homogenous cellular plasma membrane staining could be observed, which indicated membrane fusion (Figure 5A). To examine the effect of BM maturation and integrity on the treatment with FL and PEG-EL, we used flow cytometry and evaluated the relative change of median intensity of the liposomal tracer dye DiR in cells from the MCF-10A acini model after treatment with FL or PEG-EL (Figure 5C). Pretreatment of the acini with EGF triggered a significantly higher uptake of DiR upon FL treatment, demonstrated by a higher relative change of median fluorescence intensity when compared to cells from acini with a highly developed (hd) BM without EGF treatment (ld + EGF vs. hd; hd + EGF vs. hd). These influences on liposomal uptake were not observed upon treatment with PEG-EL with the same tracer dye. In addition, a significant higher relative change of fluorescence intensity was observed when the treatment with FL was compared with that with PEG-EL in cells of acini with a lowly developed BM (ld). The same held for acini with compromised basement membranes (ld + EGF; hd + EGF). In contrast, the relative change of median fluorescence showed no significant changes if a highly developed and intact BM was present (hd).

To further confirm a limiting effect of the BM on FL delivery, collagen IV was digested before treatment with FL. Confocal microscopy of collagen IV and laminin 332 clearly showed BM disruption by collagenase IV treatment (Figure 5B,D as reference). After collagenase IV treatment, the relative change of median tracer dye intensity was comparable to the relative change observed after EGF treatment. This confirmed the involvement of BM in the restriction of delivery by FL (Figure 5E). In summary, an intact, mature BM hindered liposomal uptake, while BM damage facilitated delivery, especially via FL.

### 2.4. The Anti-Proliferative Effect of DOX in 3D Breast Acini Models Depends on the Basement Membrane Status

**Figure 6:**
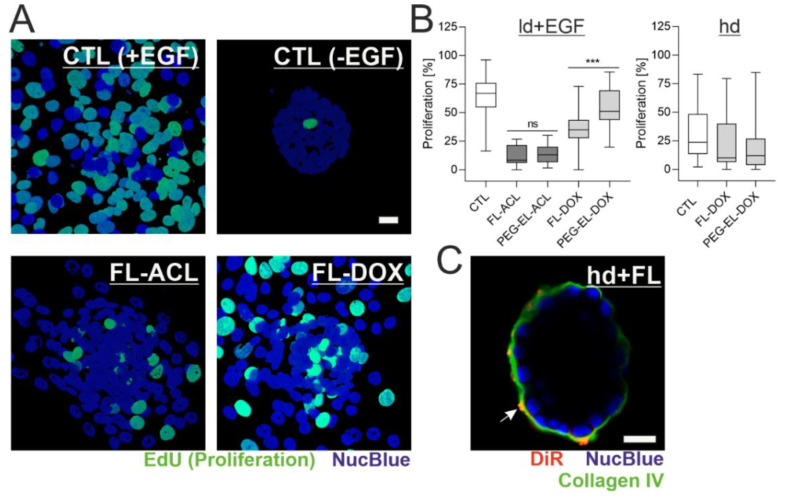
Formulation affects potency of DOX depending on the BM status. MCF-10A acini with a lowly developed and compromised BM were treated with ACL and DOX in the liposomal formulations and analyzed by the EdU incorporation assay (**A**). Quantitative analysis indicated an overall significant reduction of proliferation, except for the treatment with PEG-EL-DOX, with a significantly higher reduction when FL-DOX was compared with PEG-EL-DOX (**B**). Samples treated with PBS served as a control (CTL). This effect was not found when proliferation was investigated in MCF-10A acini with a highly developed and intact BM (hd; B). Association of the liposome dye DiR with the BM could be shown by immunofluorescent staining of collagen IV after treatment of hd-BM acini with FL (**C**). Scale bars 20 µm. Box plots are shown, statistical evaluation was done using Kruskal–Wallis test, with post-hoc Dunn’s test (***: *p* < 0.0005).

In the next step, we explored the impact of BM presence and status on the previously observed anti-proliferative effect of ACL and DOX in different liposomal formulations. As visualized with the EdU incorporation assay, the proliferation rate of MCF-10A cells in EGF-treated acini with an ld BM (ld + EGF) was 63%. These samples served as a control system. They showed a complete loss of acinar morphology and, instead, formed 2D layers of partially proliferating cells. Treatment with FL-ACL and FL-DOX led to reduced cell proliferation. In contrast, samples without EGF treatment showed almost no proliferation, therefore directly linking EGF stimulation to proliferation initiation (Figure 6A). In accordance with previous observations in 2D, the encapsulation of DOX in FL reduced proliferation significantly, whereas the encapsulation of DOX in PEG-EL did not. While, overall, all formulations besides PEG-EL-DOX led to a significant proliferation reduction compared to the control, there was no formulation-dependent proliferation difference for ACL (Figure 6B), which again confirms previous observations.

In the absence of EGF, measurements on acini with an hd BM showed that neither treatment with FL-DOX nor treatment with PEG-EL-DOX resulted in significant differences with respect to the control samples (Figure 6B). This implies that the previously observed higher impact of FL-DOX on proliferation is fully dependent on BM damage and abolished by an intact BM. Interestingly, immunofluorescent staining of the BM component collagen IV after treatment of hd-BM acini with FL suggested an interaction of DiR-containing FL with the BM, while fusion events as indicated by homogenous plasma membrane staining were rare (Figure 6C).

## 3. Discussion

In the present study, we focused on the delivery of the anthracyclines DOX and ACL using two different liposomal types, PEGylated phosphocholine-based liposomes and a novel type, fusogenic liposomes, as nano-carriers [7,18]. Previous work in traditional cell cultures has shown that PEG-EL particles are taken up by cells via endocytosis, whereas FL fuse directly with the cell membrane [18]. Liposomal uptake was investigated in a three-dimensional cell culture model of the mammary gland in which cells form acini enclosed by a BM that undergoes maturation [30].

DOX is one of the most effective anti-cancer therapeutics, commonly used to treat leukemia, lymphomas, and solid tumors [32], while ACL is a less popular chemotherapeutic, yet approved in combination with cytarabine in Japan and China [33]. ACL and DOX belong to the anthracycline family. Though the full range of pharmacological interactions of anthracyclines in cancer cells is still under discussion, interference with DNA and inhibition of the DNA–Topoisomerase II complex during DNA replication as well as production of free radicals by redox cycling are identified mechanisms for many anthracycline analogues [34,35,36]. Yet, the sensitivity of DOX-resistant tumors and cell lines to ACL indicates a difference in the mechanisms of action of the two anthracyclines [37], which is further supported by their differential intracellular localization visualized here by confocal microscopy (Figure 3). Despite potential differences in the pharmacological effects, treatment with both representatives of the anthracycline class reduces cell proliferation of fast-proliferating cancer cells, which led to the approval of many anthracyclines for the treatment of neoplastic conditions [38].

To mitigate the numerous side effects of traditional anthracyclines, they are frequently used in liposomal formulations in therapeutic applications [8,32,39,40]. In this study, we aimed to compare the effects of the encapsulation of DOX and ACL in two distinct liposomal systems. Classical phospholipid-based liposomes with a covalently bound PEG layer on the surface, based on currently approved drug formulations [7,28], were compared with fusogenic liposomes.

In order to investigate the liposomal encapsulation of ACL and DOX, we used fluorescence spectroscopy and observed a Foerster resonance energy transfer (FRET) of ACL to the liposomal tracer dye DiR in FL. Since this requires close proximity of both fluorophores, with distances of only a few nanometers [41,42], the lipophilic compound ACL might better intercalate into the bilayer of FL in comparison to PEG-EL. As earlier work pointed towards contributions of 3D lipid phases to the membranes of FL, whereas PEG-EL are formed exclusively by lipid bilayers, differences in surface hydrophilicity are expected and might explain this different behavior [43]. However, the encapsulation efficiencies of the anthracyclines in both liposomal formulations remained comparably high (Table 1), suggesting a difference in the localization of ACL in the liposome formulations rather than in the extent of encapsulation.

Differences in the anti-proliferative efficiency of DOX were detected in 2D as well as in 3D cell culture models of MCF-10A. The best efficiency could be achieved using FL as a delivery carrier (Figure 4 and Figure 6). We assume that these differences can be attributed to the action mechanism of FL. The main difference between the two formulations was found in their cellular uptakes. The classical PEGylated liposomes are usually taken up via endocytosis. Compared to this, FL are able to fuse with the plasma membrane of mammalian cells, releasing their cargo directly into the cell cytoplasm, thus completely bypassing the endosomal uptake route [17,20] and the possible degradation of the encapsulated cargo, which allows DOX to enter the cell more efficiently, enhancing its anti-proliferative effects. In the case of ACL, the positive effect of fusion in comparison to endocytosis was detectable neither in the 2D nor in the 3D cell culture model. Based on the profound difference of the liposomal uptake mechanism, we hypothesized that the drug formulation could also influence the mechanism of action of the encapsulated cargo. Yet, this hypothesis is not supported by our microscopic observations (see Supplemental Figure S1), which showed no effect of the formulation on the intracellular localizations of ACL and DOX. However, a difference in the basicity of the two anthracyclines suggests DOX to be much more sensitive to lysosomal entrapment and degradation in comparison to ACL [44,45]. Hence, the benefit of membrane fusion over endocytosis as an uptake mechanism might be more pronounced for anti-cancer agents, such as DOX, with strong lysosomotropic preferences. Here, membrane fusion can circumvent the lysosomal entrapment of DOX, enabling increased delivery and anti-proliferative effects in 2D as well as 3D for the FL formulation (Figure 4). In comparison, ACL is trapped in lysosomes at a much lower level than DOX due to its lower pKa value of 7.3 [44]. Consequently, changing the uptake route from endocytosis to fusion by varying the delivery system does not have a significant impact on the delivery efficiency of ACL.

The investigation of the cellular uptake of nano-carriers in biological systems, such as blood and body fluids, collagen matrix, or cellular tissues, is essential for future in vivo applications. In this study, a three-dimensional acini model of the mammary gland surrounded by a BM was chosen, and the liposomal uptake through this natural barrier was investigated.

BM is a known physiological barrier for the passage of macromolecules, and its dysfunction is involved in the onset of a variety of diseases [46]. This is equally important for the passage of nano-carriers such as liposomes. Here, we could examine the passage through the BM and subsequent cellular uptake of two liposomal formulations in a 3D model system consisting of cells surrounded by an endogenously formed BM. In vivo, alterations in cell–matrix interactions are closely linked to the development and progression of epithelial-derived cancers. Early lesions of the BM as well as loss of cell polarity mark the development of in situ carcinomas of the mammary gland [47], while subsequent crossing of the BM by invasive cells plays an important role in malignant transition and metastasis [48]. In this study, we modulated the BM meshwork via two routes, namely, variation of the developmental stage of the acini by duration of cultivation and EGF-induced BM disintegration, to obtain models of the transitional states of the BM in breast cancer onset. This enabled the investigation of the passage of both liposomal formulations with anti-cancer therapeutics as a cargo in a therapeutically relevant in vitro model.

BM is a highly organized and condensed extracellular matrix structure separating epithelial and stromal cells and regulating molecular diffusion to the human mammary gland tissue. BM consists of a collagen IV mesh anchored to laminins, nidogen, and perlecan [49], while heparan sulfate proteoglycans determine its negative charge [50]. To date, it is still unclear whether the BM could serve as a charge-selective barrier [50,51] and be more permeable to cationic compounds such as FL. Our microscopic study clearly underlined a charge-driven electrostatic interaction of FL with the BM surface of MCF-10A acini, not observed for negatively charged PEG-EL (see Figure 3 and Figure 6, and Supplemental Figure S3). PEGylated liposomes, in general, have been developed for prolonged circulation in body fluids, which is enabled by their reduced adsorption to serum proteins and other biological surfaces [52]. In contrast, FL exhibit a high, positive surface charge due to their high content of cationic lipids. Therefore, they are prone to strongly interact with the negatively charged BM meshwork.

Additionally to its charge-selective barrier properties, the BM meshwork acts as a molecular sieve for macromolecules [30,53,54] The permeability of this sieve is dependent on the thickness of the BM, which we controlled over the maturation time [30]. In our model, a fully mature BM (hd) was able to retain and exclude liposomes with an average size of 80–165 nm independent of nanoparticle charge. A low maturation state or a disruption of the BM clearly enhanced the efficiency of membrane fusion of cationic liposomes compared with endocytosis (Figure 5). Presumably, the charge-derived selective retention of cationic particles played the main role for this effect. When liposomes of both formulations were loaded with the anti-cancer therapeutic DOX, our observations supported the hypothesis described above. The anti-proliferative effect of DOX encapsulated in FL was much more pronounced if the basement membrane was compromised, while an intact and highly developed basement membrane attenuated this effect. While treatment of seeded cells and of the acini model with a compromised basement membrane led to significant reductions of proliferation for DOX in FL in comparison to PEG-EL, encapsulation in PEG-EL did not reduce proliferation in comparison to the control. Hence, the encapsulation of DOX in FL might optimize the drug availability at tumor sites for cells free of or beneath a thinned basement membrane, e.g., at sites of basement membrane lesion or infiltration, which are indicative of pathophysiological processes observed in cancer progression [24,55].

## 4. Material and Methods

### 4.1. Preparation of Fusogenic and Endocytic Liposomes

The lipids and lipid derivatives 1,2-dioleoyl-sn-glycero-3-phosphoethanolamine (DOPE), 1,2-dioleoyl-3-trimethylammonium-propane (DOTAP), 1,2-dipalmitoyl-sn-glycero-3-phosphocholine (DPPC), cholesterol, and 1,2-dioleoyl-sn-glycero-3-phosphoethanolamine-*N*-[methoxy-(polyethylene glycol)-2000] (PEG(2000)-PE) were purchased from Avanti Polar, Inc. (Alabaster, AL, USA) and either dissolved or readily purchased in chloroform 10 mg/mL. The carbocyanine dye DiR (ThermoFisher Scientific, Waltham, MA, USA), aclacinomycin A (Santa Cruz Biotechnology, Dallas, TX, USA), and doxorubicin hydrochloride (Sigma-Aldrich, St. Louis, MO, USA) were dissolved in chloroform at 1 mg/mL (DiR and aclacinomycin A) or in a 9/1 (*v*/*v*) mixture of chloroform and ethanol absolute (Doxorubicin hydrochloride). For the preparation of FL, DOPE and DOTAP were mixed at a mass ratio of 1/1 in chloroform, with further addition of 2 mol% DiR. For PEG-EL, the lipid mass ratio was based on the ratio of DOXIL^®^ liposomes, with addition of DiR for visualization. DPPC, PEG(2000)-PE, and cholesterol were mixed at a mass ratio of 3/1/1 under addition of 2 mol% DiR. The anthracyclines doxorubicin hydrochloride (DOX) and aclacinomycin A (ACL) were incorporated by addition at the final treatment concentrations of 0.01 µM–15 µM. Free drugs without lipid served as controls.

Lipid films with or without anthracyclines were generated by evaporation of the solvents in vacuum overnight. They were hydrated with 20 mM 2-(4-(2-hydroxyethyl)-1-piperazinyl)-ethanesulfonic acid buffer (VWR, Darmstadt, Germany) in purified, degassed, and filtrated water (Milli-Q, Merck Millipore, Darmstadt, Germany) at pH 7.4, at a total lipid concentration of 2 mg/mL. FL and PEG-EL solutions were then sonicated in an ultrasonic bath (Sonocool SC 255; Bandelin, Berlin, Germany) for 20 min at 4 °C. Liposomes were kept at 4 °C until usage and were not stored longer than 24 h.

### 4.2. Characterization of Liposomes

The size and zeta potential distributions of both liposomal formulations with or without anthracyclines were evaluated by dynamic and electrophoretic light scattering, respectively, using a Nano ZS Zetasizer (Malvern Instruments, Malvern, UK). Samples were diluted 1:10 in purified, degassed, and filtrated water (Milli-Q, Merck Millipore, Darmstadt, Germany) for size distribution measurements and 45-fold for zeta potential distributions. Measurements were performed on at least three independently prepared solutions.

For the measurement of fluorescence, samples were diluted 1:50 (*v*/*v*) in phosphate-buffered saline (PBS; 137 mM NaCl, 2.7 mM KCl, 10 mM Na_2_HPO_4_, 1.8 mM KH_2_PO_4_, pH 7.4) and measured in a spectrometer (Fluorolog-3, Horiba Jobin Yvon, Kyoto, Japan). Briefly, emission spectra were detected by excitation at 488 nm or 633 nm and recorded at 510–900 nm or 700–900 nm, respectively. Excitation spectra were recorded at a constant emission at 800 nm and an excitation range of 600 nm–790 nm. Excitation/emission slit widths and integration time were kept equal and constant during the measurements of DiR, ACL, and DOX.

Analysis of the encapsulation efficiency (EE) of ACL and DOX in FL and EL was modified from a previously published method [56]. Briefly, both formulations loaded with the anthracyclines were centrifuged in Nanosep^®^ centrifugal devices with a 100 K Omega™ Membrane (Pall, NY, USA) for 6 min at 12,000× *g* and 4 °C. The fraction of unbound DOX was determined from the flowthrough (ft) using a calibration curve of free DOX and subsequent fluorescence spectroscopy, as described above (see Equation (1)). The fraction of unbound ACL was determined after elution (el) of the more lipophilic anthracycline from the filter membrane using 0.5% BSA in PBS with a calibration curve of free ACL and subsequent fluorescence spectroscopy, as described above (see Equation (2)).

(1) $$EE\;DOX\left(\%\right)=100\%-DOX_{\text{ft}}\left(\%\right)$$

(2) $$EE\;ACL\left(\%\right)=100\%-ACL_{\text{el}}\left(\%\right)$$

### 4.3. Cell Maintenance and Cultivation of MCF-10A Acini

Experiments were performed on the MCF-10A cell line purchased from ATCC (Manassas, VA, USA). Cells were cultured in 2D in DMEM/F12 (Life Technologies, Carlsbad, CA, USA) containing 5% horse serum (New Zealand origin, Life Technologies, Carlsbad, CA, USA), 20 ng/mL EGF (Sigma-Aldrich, St. Louis, MO, USA), 0.5 μg/mL hydrocortisone, 100 ng/mL cholera toxin, 10 μg/mL insulin, 100 U/mL penicillin/streptomycin (all Sigma-Aldrich). Acini from MCF-10A were cultured as previously described [30]. In brief, single cells were seeded onto a Geltrex (ThermoFisher Scientific, Waltham, MA, USA) bed and cultivated for nine days in EGF-supplemented assay medium (DMEM/F12 Glutamax, 2% horse serum, 5 ng/mL EGF (Sigma-Aldrich, St. Louis, MO, USA), 0.5 μg/mL hydrocortisone, 1 ng/mL cholera toxin, 10 μg/mL insulin, 100 U/mL penicillin/streptomycin), changing the medium every three days. At day nine, EGF was eliminated from the assay medium for further cultivation, changing the medium every three days [30], while acini cultivation did not exceed 21 days.

### 4.4. Liposomal Treatment and Imaging of Cellular Uptake in 2D

For the treatment of MCF-10A cells with FL or PEG-EL, the liposomal formulations with or without anthracyclines were diluted 1:50 (*v*/*v*) with PBS and incubated with cells seeded on a high-precision microscopy glass (ThermoFisher Scientific, Waltham, MA, USA) for 10 min at 37 °C and 5% CO_2_. After washing with PBS, cells were either covered with cell culture medium and imaged live or fixed with 3.7% paraformaldehyde and stained with NucBlue for fixed cells (ThermoFisher Scientific, Waltham, MA, USA). The confocal laser scanning microscope LSM880 equipped with a C-Apochromat 40×/1.2 W objective was used for imaging (all Carl Zeiss, Jena, Germany). The anthracyclines ACL and DOX were imaged by excitation with an argon ion laser at 488 nm and an emission bandwidth of 499–597 nm. The dye DiR was imaged by excitation at 633 nm and an emission bandwidth of 651–758 nm. Data with anthracyclines were presented as maximum intensity projections of z-stacks for better visualization of the anthracycline and fusion (i.e., DiR) signal. Further, the incorporation of the membrane tracer dye DiR after treatment with both liposomal formulations was examined. Here, NucBlue was imaged by excitation at 405 nm and an emission bandwidth of 446–499 nm for nucleus tracking in addition to DiR imaging.

### 4.5. Measurement of the Anti-Proliferative Effects of the Anthracyclines DOX and ACL

#### 4.5.1. Cell Counting Using Flow Cytometry

The anti-proliferative effect of the anthracyclines DOX and ACL in FL was investigated using flow cytometry. Anthracyclines were serially diluted from 15/3 µM to 0.01 µM as the final treatment concentration in FL. MCF-10A cells were treated for 10 min at 37 °C and 5% CO_2_, followed by a washing step with PBS and subsequent reseeding of the cells. After an additional incubation period of 72 h at 37 °C and 5% CO_2_ to promote cell proliferation, the cells were detached with 0.05% Trypsin/EDTA (Life Technologies, Carlsbad, CA, USA), and the cell suspension was subjected to flow cytometry (Guava easyCyte 8HT; Merck Millipore, Burlington, VT, USA). Briefly, the cell population was identified in forward and side scatter by identical population gating, and cell numbers were evaluated by the manufacturer’s software.

#### 4.5.2. EdU (5-ethynyl-2′-deoxyuridine) Incorporation Assay

To compare the different formulations of anthracyclines in two-dimensional (2D) cell culture and the three-dimensional (3D) MCF-10A acini model, an EdU incorporation assay was used. MCF-10A cells were seeded on a high-precision microscopy glass, whereas acini were transferred after 10 days or 20 days of cultivation on a high-precision microscopy glass coated by a thin layer of Geltrex, incubated with or without EGF, respectively. As described previously, after day 10 or 20, MCF-10A acini represent low- and high-maturation states of the basement membrane, respectively [30,31]. 2D samples were treated with PBS-diluted liposomal formulations or free drugs for 10 min at 37 °C and 5% CO_2_, with final concentrations of 1.8 µM DOX or 0.6 µM ACL in the treatment solution. After treatment, the samples were washed with PBS and further cultured in cell culture medium supplemented with 10 µM EdU (5-ethynyl-2′-deoxyuridine) to monitor the proliferation of the cells in this time frame. The treatment period for the 3D samples was extended to 30 min at 37 °C and 5% CO_2_ to enable permeation through the basement membrane. Medium for further cultivation was supplemented with EdU and EGF after treatment. All samples were fixed 72 h after cell seeding or acini transfer and coupled with an Alexa Fluor 488 fluorophore according to the manufacturer’s instructions (Click-it EdU Alexa Fluor 488 Imaging Kit, ThermoFisher Scientific, Waltham, MA, USA), followed by nuclei counterstaining with NucBlue stain for fixed cells (ThermoFisher Scientific, Waltham, MA, USA) at RT. Imaging was done with the same microscope as described above, using the Airyscan detector to improve resolution. Alexa Fluor 488 coupled to EdU incorporated in the newly synthesized DNA of proliferating cells was excited at 488 nm; emission was detected with a bandpass filter (495–550 nm). The NucBlue signal of all cell nuclei was excited at 405 nm, and emission was detected with another bandpass filter (420–480 nm). While seeded cells were imaged in a focal plane, imaging of acini required confocal image stacks. The analysis of the recorded microscopy data and nucleus count was carried out after randomization using Matlab R2019b (MathWorks, Natick, MA, USA) for 2D and 3D samples. To quantify NucBlue- and/or proliferation-positive nuclei in 2D samples, count of focal plane images was done manually using Fiji-ImageJ [57]. For 3D acini samples, nuclei count was done semi-automatically using Imaris 9.5.0 (Bitplane, Zürich, Switzerland). The percentage of double-positive nuclei is shown as the proliferation fraction in both cases.

### 4.6. Evaluation of Membrane Tracer Dye DiR Uptake in Cells of the MCF-10A Acini Model

Acini with a lowly or highly developed basement membrane were transferred onto high-precision glass and either directly treated or incubated for another 30 h with addition of 20 ng/mL EGF to promote cell proliferation and loss of acinar polarization [30]. For liposome treatment, the liposome suspension was diluted 1:50 (*v/v*) in PBS and incubated for 30 min at 37 °C and 5% CO_2_, followed by a PBS washing step.

For observation of DiR incorporation, nuclei were counterstained with NucBlue prior to imaging. The incorporation of the membrane tracer dye DiR was imaged with the confocal laser scanning microscope described above and excitation at 633 nm, with emission bandwidth of 651–758 nm for membrane tracer dye visualization. Nuclei were visualized by excitation at 405 nm with emission bandwidth of 410–499 nm. For basement membrane visualization, acini were transferred and either directly fixed or treated with 290 U/mL collagenase IV (Worthington Biochemical Coorporation, Lakewood, NJ, USA) with subsequent fixation, followed by immunostaining with the primary antibodies anti-laminin-5 (clone D4B5; Merck Millipore, Burlington, VT, USA; note that in modern nomenclature, laminin-5 is called laminin 332) and/or anti-type IV collagen (polyclonal; Abcam, Cambridge, UK), followed by subsequent coupling with fluorophore-linked secondary antibodies (donkey anti-mouse Alexa Fluor 546 and goat anti-rabbit Alexa Fluor 488, respectively, both Life Technologies, Carlsbad, CA, USA) and nucleus counterstaining. Collagen IV and laminin 332 were visualized by excitation at 488 nm and 561 nm wavelength and emission bandwidth of 499–597 nm and 588–695 nm, respectively.

For quantification of the membrane tracer dye uptake, basement membrane and cell cluster were dissolved by treatment with 290 U/mL collagenase IV and 0.04% trypsin/EDTA for 20 min at 37 °C and 5% CO_2_ to obtain a single-cell suspension after liposome treatment. Treatment with FL after collagenase IV but before trypsin/EDTA treatment served as a fusion control of a highly developed, but digested, basement membrane. The relative change of median DiR intensity of the resulting single-cell suspensions, fixed with solution A (10%) from the fix and perm cell fixation and cell permeabilization kit (ThermoFisher Scientific, MA, USA), was monitored using flow cytometry. Using the Guava easyCyte 8HT (Merck Millipore, Burlington, VT, USA), single-cell suspensions were excited with a 635 nm laser, and fluorescence emission of DiR was detected in a 785/70 nm band-pass optical filter, after identification of the cell population using forward and side scatter and subsequent doublet exclusion using forward scatter height and area. Using the CytoFlex S (Beckman Coulter, Brea, CA, USA), the single-cell suspensions were excited with a 638 nm laser, and fluorescence emission of DiR was detected in a 780/60 nm band-pass optical filter, after identification of the cell population using forward and side scatter and subsequent doublet exclusion using forward scatter height and area. The ratio of sample median fluorescence intensity to control median fluorescence intensity was examined in an identical procedure and is shown as relative change of the median shift.

### 4.7. Data Presentation and Statistical Analysis

Data were analyzed and presented using GraphPad Prism 7 (GraphPad Software Inc, San Diego, CA, USA), BioDraw Ultra 12.0 (CambridgeSoft, Cambridge, MA, USA), Fiji-ImageJ [57], Imaris 9.5.0 (Bitplane, Zürich, Switzerland), Matlab R2019b (MathWorks, Natick, MA, USA) and CorelDRAW 2019 (Corel, Ottawa, ON, Canada). Brightness and contrast of micrographs were enhanced for visualization. Statistical analysis was carried out on data of at least three individual experiments and presented as mean with confidence interval (95%) unless indicated otherwise, followed by analysis with parametric one-way ANOVA followed by Tukey’s post-hoc test. If D’Agostino–Pearson normality test failed (alpha < 0.05), data were analyzed with non-parametric Kruskal–Wallis test followed by Dunn’s post-hoc test.

## 5. Conclusions

In this study, we demonstrated the delivery of cargo by two types of liposomes, PEGylated phosphocholine-based liposomes and fusogenic liposomes. The comparison of membrane fusion and endocytosis as uptake mechanism suggested an advantage for FL in the delivery of cargo that is sensitive for lysosomal entrapment and degradation. Further, delivery by FL appeared to be modulated by the endogenously derived basement membrane covering targeted epithelial cells in a 3D breast acini model. Here, increasing the thickness of the basement membrane reduced the delivery of a tracer dye in FL as well as the anti-proliferative efficiency of the anti-cancer therapeutic DOX serving as a cargo, while integrity loss of the basement membrane, as can be observed in breast cancer progression, reversed this effect. We therefore propose FL as basement membrane-selective, highly efficient nano-carriers for lysosome-sensitive cargos, on the basis of our observations of the delivery of DOX in 2D and a 3D breast acini model with modular basement membrane, indicating promising results for further medical applications.

## Figures and Tables

**Table 1:** Size and zeta potential characterization of fusogenic liposomes (FL) and polyethylene glycol (PEG)ylated endocytic liposomes (PEG-EL) loaded with the anthracyclines ACL and DOX and corresponding encapsulation efficiencies (EE) determined by filtration. Mean with standard deviation is shown.

	Size (nm)	ζ Potential (mV)	EE (%)
FL	116 ± 45	70 ± 6	-
FL-ACL	87 ± 14	64 ± 25	92 ± 6
FL-DOX	85 ± 4	69 ± 5	88 ± 6
PEG-EL	163 ± 78	−29 ± 18	-
PEG-EL-ACL	152 ± 46	−47 ± 5	89 ± 4
PEG-EL-DOX	142 ± 44	−48 ± 10	90 ± 6

## Supplementary Materials

The following are available online at https://www.mdpi.com/1424-8247/13/9/256/s1, Figure S1: Localization of anthracyclines ACL and DOX in MCF-10A cells, Figure S2: Anti-proliferative effect of the anthracyclines ACL and DOX upon delivery via PEG-EL and as free drug, Figure S3: Interaction of fusogenic liposomes and endocytic liposomes with the surface of acini with a lowly developed basement membrane.

## Abbreviations

Aclacinomycin a: ACL; basement membrane: BM; bovine serum albumin: BSA; doxorubicin: DOX; elute: el; encapsulation efficiency: EE; epidermal growth factor: EGF; flowthrough: ft; Foerster resonance energy transfer: FRET; fusogenic liposomes: FL; highly developed: hd; lowly developed: ld; phosphate-buffered saline: PBS; polyethylene glycol: PEG; PEGylated liposomes: PEG-EL; two-dimensional: 2D; three-dimensional: 3D.
