# Supplementary material for: The Basement Membrane in a 3D Breast Acini Model Modulates Delivery and Anti-Proliferative Effects of Liposomal Anthracyclines
Source: Pharmaceuticals (Basel). 2020 Sep 19;13(9):256. doi: 10.3390/ph13090256 (PMC7558514; doi:10.3390/ph13090256)
Supplement: Supplementary file 1 [file pharmaceuticals-13-00256-s001.pdf]

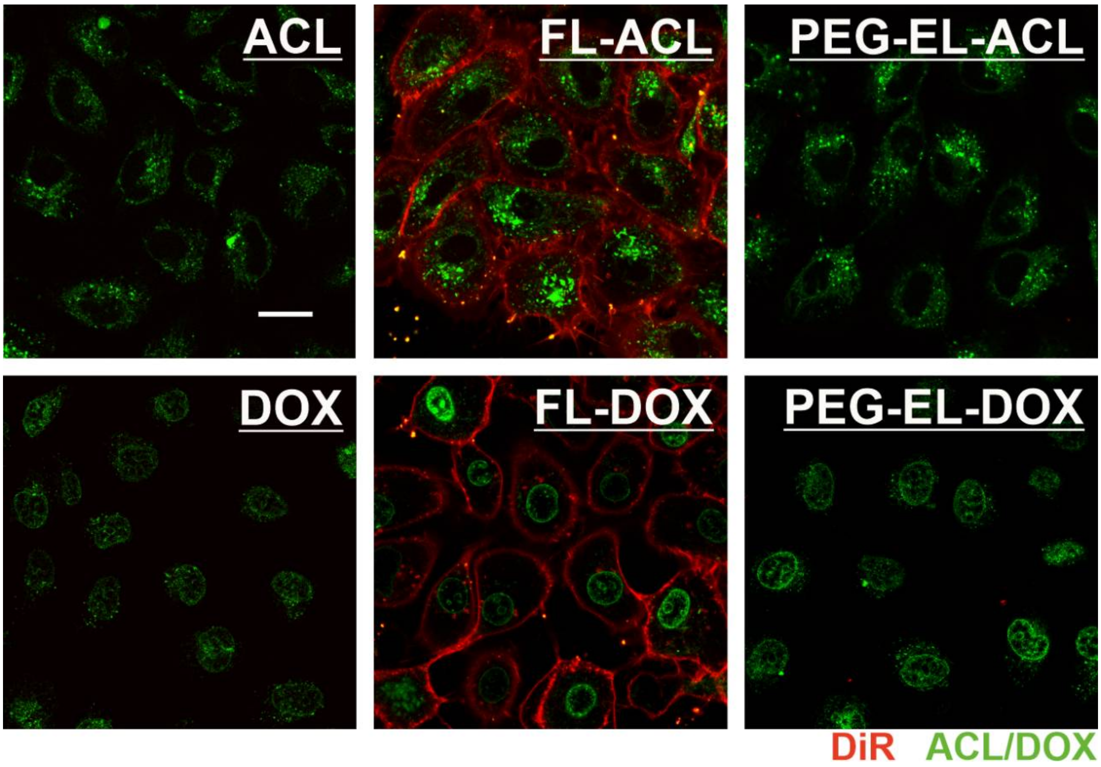

Supplemental Figure S1. Localization of anthracyclines ACL (top row) and DOX (bottom row) in MCF-10A cells. Incorporation of the free drugs (ACL, DOX), the drugs encapsulated in FL (FL-ACL, FL-DOX) and in PEG-EL (PEG-EL-ACL, PEG-EL-DOX) resulted in similar localization. Scale bar 20 μm, applies to all.

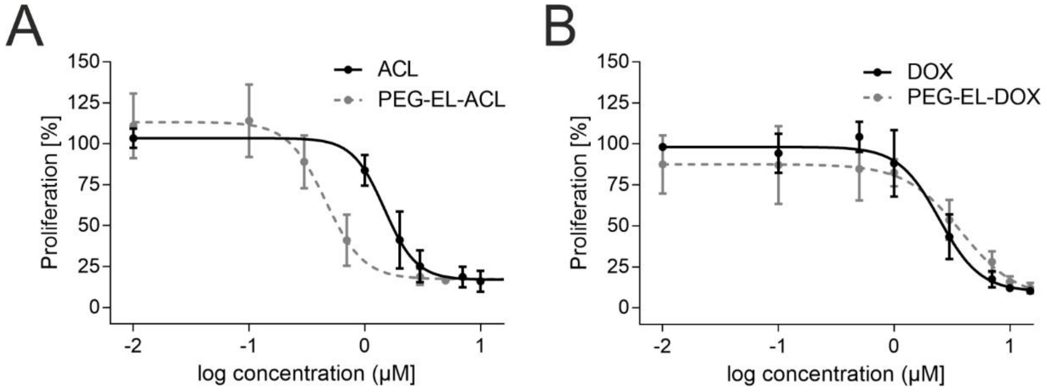

Supplemental Figure S2. Anti-proliferative effect of the anthracyclines ACL (A) and DOX (B) upon delivery via PEG-EL and as free drug. ACL is more potent than DOX and the effect is dose-dependent. Mean with standard deviation is shown.

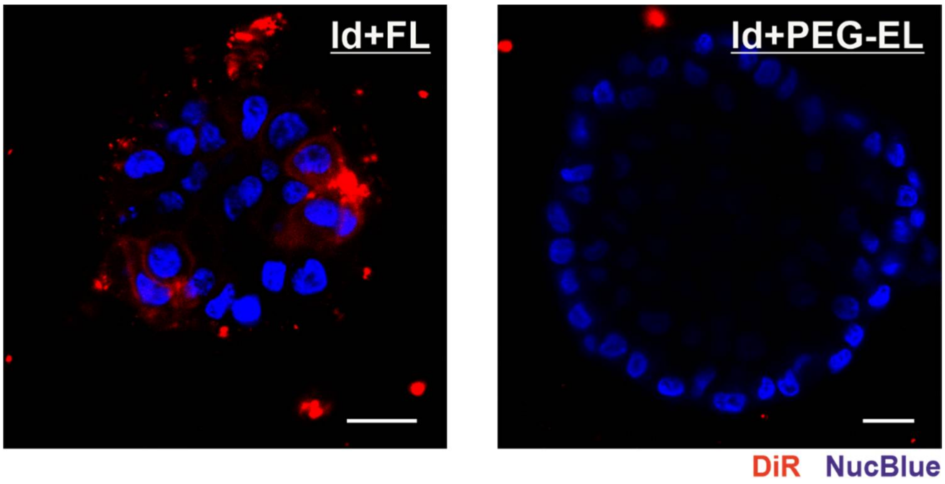

Supplemental Figure S3. Interaction of fusogenic liposomes (FL) and endocytic liposomes (PEG-EL) with the surface of acini with a lowly developed basement membrane (ld). Scale bars 20 μm.
